# Comparative analysis and characterization of the chloroplast genome of *Krascheninnikovia ceratoides* (Amarathaceae): a xerophytic semi-shrub exhibiting drought resistance and high-quality traits

**DOI:** 10.1186/s12863-024-01197-y

**Published:** 2024-01-29

**Authors:** Yuping Liu, Changyuan Zheng, Xu Su, Jinyuan Chen, Xiaoli Li, Chenglin Sun, Mir Muhammad Nizamani

**Affiliations:** 1 School of Life Sciences, Qinghai Normal University, 38# Wusixi Road, Xining, 810008 Qinghai Province China; 2 Academy of Plateau Science and Sustainability, Qinghai Normal University, 38# Wusixi Road, Xining, 810008 Qinghai Province China; 3 Key Laboratory of Biodiversity Formation Mechanism and Comprehensive Utilization of Qinghai-Tibetan Plateau in Qinghai Province, Qinghai Normal University, 38# Wusixi Road, Xining, 810008 Qinghai Province China; 4 Department of Plant Pathology, Agricultural College, Guizhou University, Guiyang, Guizhou China

**Keywords:** Chloroplast genome, Codon usage, Gene loss event, *Krascheninnikovia ceratoides*, Phylogenetic analysis, Simple repeat sequence

## Abstract

**Background:**

*Krascheninnikovia ceratoides*, a perennial halophytic semi-shrub belonging to the genus *Krascheninnikovia* (Amarathaceae), possesses noteworthy ecological, nutritional, and economic relevance. This species is primarily distributed across arid, semi-arid, and saline-alkaline regions of the Eurasian continent, encompassing Inner Mongolia, Xinjiang, Qinghai, Gansu, Ningxia, and Tibet.

**Results:**

We reported the comprehensive chloroplast (cp) genome of *K. ceratoides*, characterized by a circular conformation spanning 151,968 bp with a GC content of 36.60%. The cp genome encompassed a large single copy (LSC, 84,029 bp), a small single copy (SSC, 19,043 bp), and a pair of inverted repeats (IRs) regions (24,448 bp each). This genome harbored 128 genes and encompassed 150 simple sequence repeats (SSRs). Through comparative analyses involving cp genomes from other Cyclolobeae (Amarathaceae) taxa, we observed that the *K. ceratoides* cp genome exhibited high conservation, with minor divergence events in protein-coding genes (PCGs) *accD, matK, ndhF, ndhK, ycf1,* and *ycf2*. Phylogenetic reconstructions delineated *K. ceratoides* as the sister taxon to *Atriplex, Chenopodium, Dysphania,* and *Suaeda*, thus constituting a robust clade. Intriguingly, nucleotide substitution ratios (*Ka/Ks*) between *K. ceratoides* and *Dysphania* species for *ycf1* and *ycf2* genes surpassed 1.0, indicating the presence of positive selection pressure on these loci.

**Conclusions:**

The findings of this study augment the genomic repository for the Amarathaceae family and furnish crucial molecular instruments for subsequent investigations into the ecological adaptation mechanisms of *K. ceratoides* within desert ecosystems.

**Supplementary Information:**

The online version contains supplementary material available at 10.1186/s12863-024-01197-y.

## Background

Drought stress is one of the most significant limiting factors affecting plant growth, development, and agricultural productivity worldwide [1]. As climate change progresses, the increasing frequency and severity of droughts present substantial challenges to global food security and ecosystems [2]. To mitigate the impacts of drought on agriculture, it is essential to identify and utilize drought-resistant plant species that can maintain productivity under water-limited conditions [3]. Xerophytic plants, which are adapted to arid environments, have evolved a range of morphological, physiological, and molecular adaptations that enable them to with-stand water scarcity [4].

These adaptations allow xerophytes to survive and even thrive in environments where other plant species may struggle or perish [5]. Identifying and characterizing the genetic components underlying these drought-resistant adaptations in xerophytic plants can inform plant breeding programs aiming to improve drought resistance in crop plants [6]. By incorporating these adaptive traits into crops, it may be possible to enhance their resilience to drought stress and maintain agricultural productivity in the face of an increasingly uncertain climate [7]. As a result, studying xerophytic plants and their unique adaptations to water scarcity can provide valuable insights for developing more resilient agricultural systems and ensuring global food security.

*Krascheninnikovia ceratoides*, belonging to the Amarathaceae family, is a perennial semi-shrub that thrives in arid, semi-arid, and saline-alkaline environments found in western China regions such as Inner Mongolia, Xinjiang, Qinghai, Gansu, Ningxia, and Tibet, as well as neighboring areas across the Eurasian continent [8–10]. This plant species plays a vital ecological role in desert ecosystems and is extensively utilized for local forage and economic benefits [11]. While numerous studies have delved into its ecological, physiological [12], anatomical, genetic, and chemical aspects as a member of the *Krascheninnikovia* genus, its cp genome remains unexamined. Moreover, no phylogenetic analyses based on entire cp genome have been published to date.

Prior research on *K. ceratoides* has primarily focused on its ecological and physiological traits [12–15], anatomical structure of vegetative organs [16], chromosome karyotype [17], application potential [18], chemical components [19], and genetic diversity [20]. For instance, Han et al. [20] used the RAPD (Random Amplified Polymorphic DNA) technique to investigate seven *Krascheninnikovia species*, uncovering significant distinctions and a wealth of genetic diversity among these species. Nonetheless, the cp genome of *K. ceratoides* has not yet been characterized, and no published phylogenetic analyses based on entire cp genome are available. This study intends to fill this knowledge gap by providing an in-depth understanding of the genetic makeup of this crucial plant species. By examining the cp genome of *K. ceratoides* and carrying out phylogenetic analyses with the whole cp genome, we aim to further our comprehension of the *Krascheninnikovia* genus’s evolution and diversity while supplying a valuable resource for future ecological and utilization research on *K. ceratoides*.

The influx of cp genome sequences has led to the development of advanced bioinformatics tools and resources, which are essential for analyzing and interpreting the vast amounts of data generated by next-generation sequencing (NGS) [21]. These tools include algorithms for sequence assembly, annotation, and comparative analysis, which facilitate the identification of structural variations, gene content, and functional elements within cp genomes [22]. Additionally, these tools enable researchers to investigate phylogenetic relationships among plant species and trace the evolutionary history of cp genomes [23]. The expansion of cp genome data has significantly broadened our knowledge of cp genome diversity and evolution [24]. This wealth of information has unveiled unique features, such as variations in gene content, gene order, and repeat elements, which offer insights into the adaptive processes and evolutionary forces that have shaped cp genomes over time [25, 26]. Furthermore, it has highlighted the potential role of cp genomes in plant speciation, adaptation, and population genetics.

Harnessing the potential of cp genetic resources has opened up new avenues in plant breeding, biotechnology, and conservation efforts [27]. In plant breeding, cp genomes can serve as a valuable source of genetic markers, which can be employed for the development of improved crop varieties with desirable traits, such as increased yield, resistance to pests and diseases, and enhanced stress tolerance [28]. In biotechnology, cp genomes offer opportunities for the engineering of transplastomic plants, which can produce high levels of bioactive compounds, biopharmaceuticals, or functional proteins for industrial, pharmaceutical, or agricultural applications [29]. Lastly, the comprehensive knowledge of cp genome diversity and evolution can aid in the conservation of plant biodiversity by providing essential information for the identification of endangered species, the assessment of genetic diversity within and among populations, and the formulation of effective conservation strategies [30]. The rapid and cost-effective sequencing of complete cp genomes, enabled by next-generation sequencing technologies and bioinformatics tools, has significantly expanded our understanding of cp genome diversity and evolution [31]. This increased knowledge has un-locked new possibilities in plant breeding, biotechnology, and conservation efforts, ultimately contributing to global food security, environmental sustainability, and human well-being.

In this study, we present a comprehensive analysis and characterization of the cp genome of *K. ceratoides*. Our objectives were to: (1) assemble and annotate the cp genome of *K. ceratoides*, (2) perform comparative analyses with related species, (3) investigate SSRs, long repeats, and codon usage patterns, and (4) elucidate the phylogenetic relationships among species in the Amarathaceae family. This information will contribute to our understanding of the genetic basis underlying the drought resistance and high-quality traits of *K. ceratoides* and inform future breeding and conservation efforts. Our results will not only contribute to the development of molecular markers for more extensive Amarathaceae investigations but also offer a theoretical foundation for genetic breeding, population research, and the evaluation of phylogenetic connections within the Amarathaceae family.

## Results

### Chloroplast genome characteristics of *Krascheninnikovia ceratoides*

**Fig. 1:**
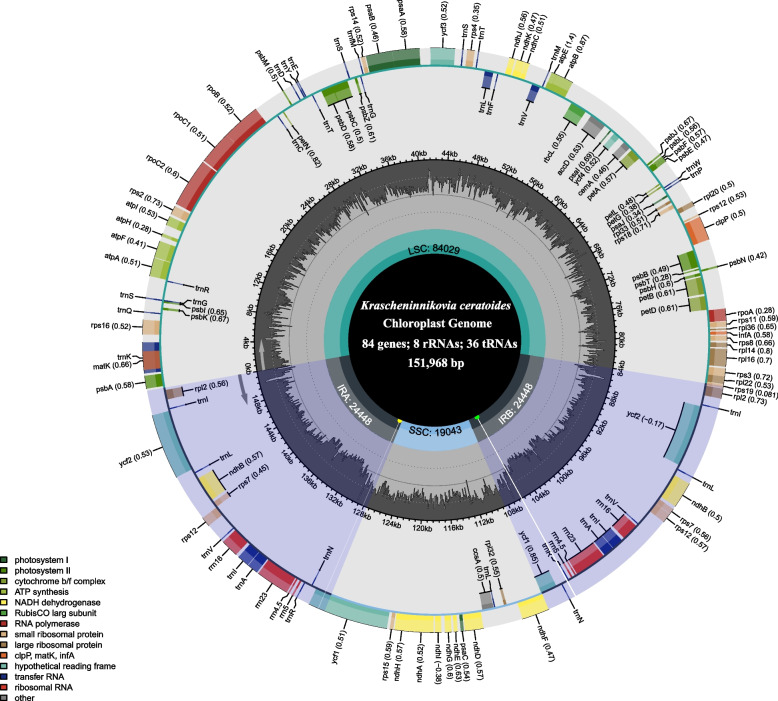
Circular map of *Krascheninnikovia ceratoides* (Amarathaceae) cp genome displays genes on the outer and inner parts of the circle, which are transcribed in counterclockwise and clockwise directions. The gray bars in the inner circle correspond to GC content. The gene names and their codon usage bias are labeled on the outermost layer

The *K. ceratoides* cp genome exhibits a typical quadripartite circular structure with a total length of 151,968 bp. This genome is composed of an 84,029 bp LSC region and a 19,043 bp small single copy (SSC) region, separated by a pair of 24,448 bp long IRa and IRb regions. The cp genome’s overall GC content is 36.60%, with the LSC, SSC, and the two IR regions having GC contents of 34.80, 30.60, and 42.20%, respectively (Table 1). The *K. ceratoides* cp genome encodes a total of 128 genes, including 84 PCGs, 36 tRNA genes, and eight rRNA genes (Table 1). Among these genes, 84 are located in the LSC region, 13 in the SSC region, and 31 in the IR regions (Fig. 1 and Table 2). Seven PCGs (*petB*, *petD*, *atpF*, *ndhA*, *rpoC1*, *rps16*, *rpl16*) and six tRNA genes (*trnA-UGC*, *trnI-CAU*, *trnK-UUU*, *trnL-UAA*, *trnR-ACG*, *trnV-UAC*) contain one intron, while three PCGs (*ndhB*, *rps12*, *clpP*) and one gene of unknown function (*ycf3*) have two introns (Tables 1 and 2).

**Table 1:** Characteristics of the chloroplast genome of *Krascheninnikovia ceratoides*

Category	Item	Characteristics of chloroplast genome
Construction of cp genome	Size of chloroplast genome (bp)	151,968
	Size of LSC (bp)	84,029
	Size of SSC (bp)	19,043
	Size of IRA (bp)	24,448
	Size of IRB (bp)	24,448
Gene content	No. of overall genes	128
	No. of protein-coding genes	84
	No. of tRNA genes	36
	No. of rRNA genes	8
	No. of duplicate genes	17
GC content	Overall GC content (%)	36.60
	GC content of LSC region (%)	34.80
	GC content of SSC region (%)	30.60
	GC content of IRA region (%)	42.20
	GC content of IRB region (%)	42.20

*LSC* Large single copy region, *SSC* Small single copy region, *IR* Inverted repeat

**Table 2:** Functional categories for the genes present in the chloroplast genome of *Krascheninnikovia ceratoides*

Category for genes	Group of genes	Name of genes
Genes for photosynthesis	Subunits of photosystem I	*psaA*, *psaB*, *psaC*, *psaI*, *psaJ*
	Subunits of photosystem II	*psbA*, *psbB*, *psbC*, *psbD*, *psbE*, *psbF*, *psbH*, *psbI*, *psbJ*, *psbK*, *psbL*, *psbM*, *psbN*, *psbT*, *psbZ*
	Subunit of cytochrome b/f complex	*PetA*, *petB*^*a*^, *petD*^*a*^, *petG*, *petN*, *petL*
	Subunits of ATP synthase	*atpA*, *atpB*, *atpE*, *atpF*^*a*^, *atpH*, *atpI*
	Subunits of NADH dehydrogenase	*ndhA*^*a*^, *ndhB*^*bc*^, *ndhC*, *ndhD*, *ndhE*, *ndhF*, *ndhG*, *ndhH*, *ndhI*, *ndhJ*, *ndhK*
	Large subunit of rubisco	*rbcL*
Self-replication	DNA dependent RNA polymerase	*rpoA*, *rpoB*, *rpoC1*^*a*^, *rpoC2*
	Small subunit of ribosome	*rps2*, *rps3*, *rps4*, *rps7*^*c*^, *rps8*, *rps11*, *rps12*^*bc*^, *rps14*, *rps15*, *rps16*^*a*^, *rps18*, *rps19*
	Large subunit of ribosome	*rpl2*^*c*^, *rpl14*, *rpl16*^*a*^, *rpl20*, *rpl22*, *rpl32*, *rpl33*, *rpl36*
	Transfer RNA genes	*trnA-UGC*^*ac*^, *trnC-GCA*, *trnD-GUC*, *trnE-UUC*, *trnF-GAA*, *trnG-GCC*, *trnG-UCC*, *trnI-CAU*^*ac*^, *trnI-GAU*^*c*^, *trnK-UUU*^*a*^, *trnL-CAA*^*c*^, *trnL-UAA*^*a*^, *trnL-UAG*, *trnfM-CAU*, *trnM-CAU*, *trnN-GUU*^*c*^, *trnP-UGG*, *trnQ-UUG*, *trnR-ACG*^*ac*^, *trnR-UCU*, *trnS-GGA*, *trnS-GCU*, *trnS-UGA*, *trnT-GGU*, *trnT-UGU*, *trnV-GAC*^*c*^, *trnV-UAC*^*a*^, *trnW-CCA*, *trnY-GUA*
	Ribosomal RNA genes	*rrn*4.5^c^, *rrn*5^c^, *rrn*16^c^, *rrn*23^c^
	Translation initiation factor	*infA*
Other genes	Maturase	*matK*
	Envelop membrane protein	*cemA*
	c-type eytochrome synthesis gene	*ccsA*
	Submit of acetyl-CoA-carboxylase	*accD*
	ATP-dependent protease subunit P	*clpP*^*b*^
Genes of unknown function	Conserved open reading frame	*ycf1*^*c*^, *ycf2*^*c*^, *ycf3*^*b*^, *ycf4*

^a^ and ^b^ represent an intron and two introns in protein-coding genes, respectively. ^c^ represents duplicated genes

### Repeat sequence and codon usage analysis in *K. ceratoides*’ chloroplast genome

**Fig. 2:**
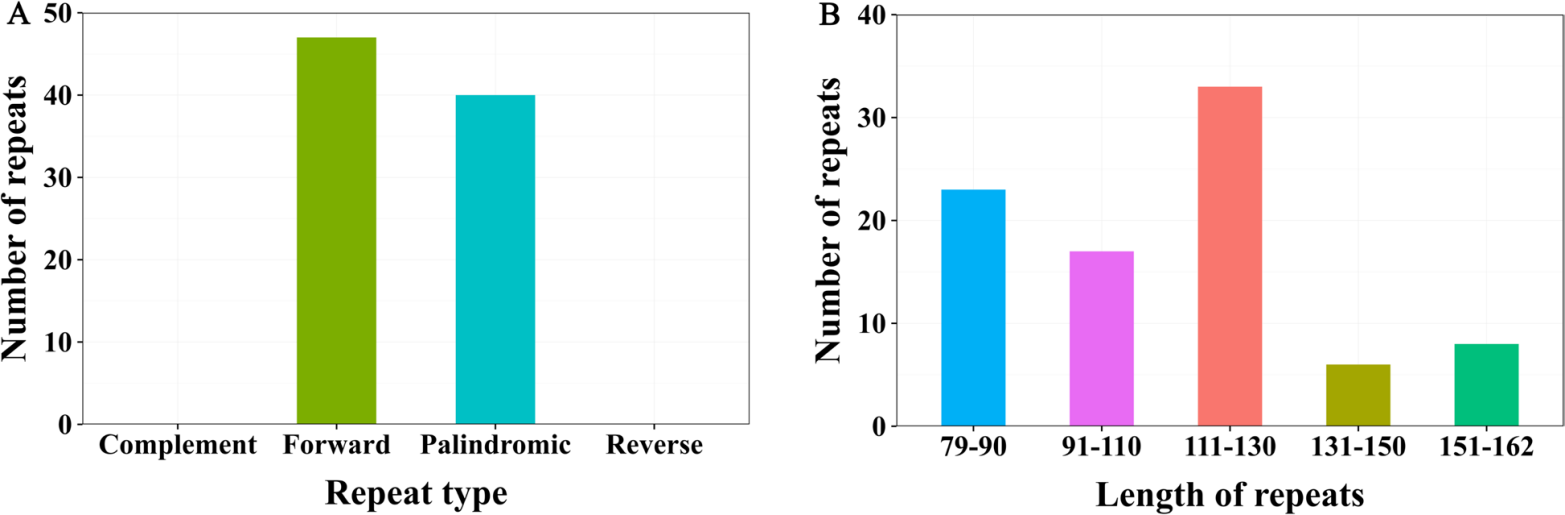
The variety and length of repeats in the *Krascheninnikovia ceratoides* cp genome are as follows: **A** The count of different types of repeat sequences; **B** The total number of repeat sequences according to their length

**Fig. 3:**
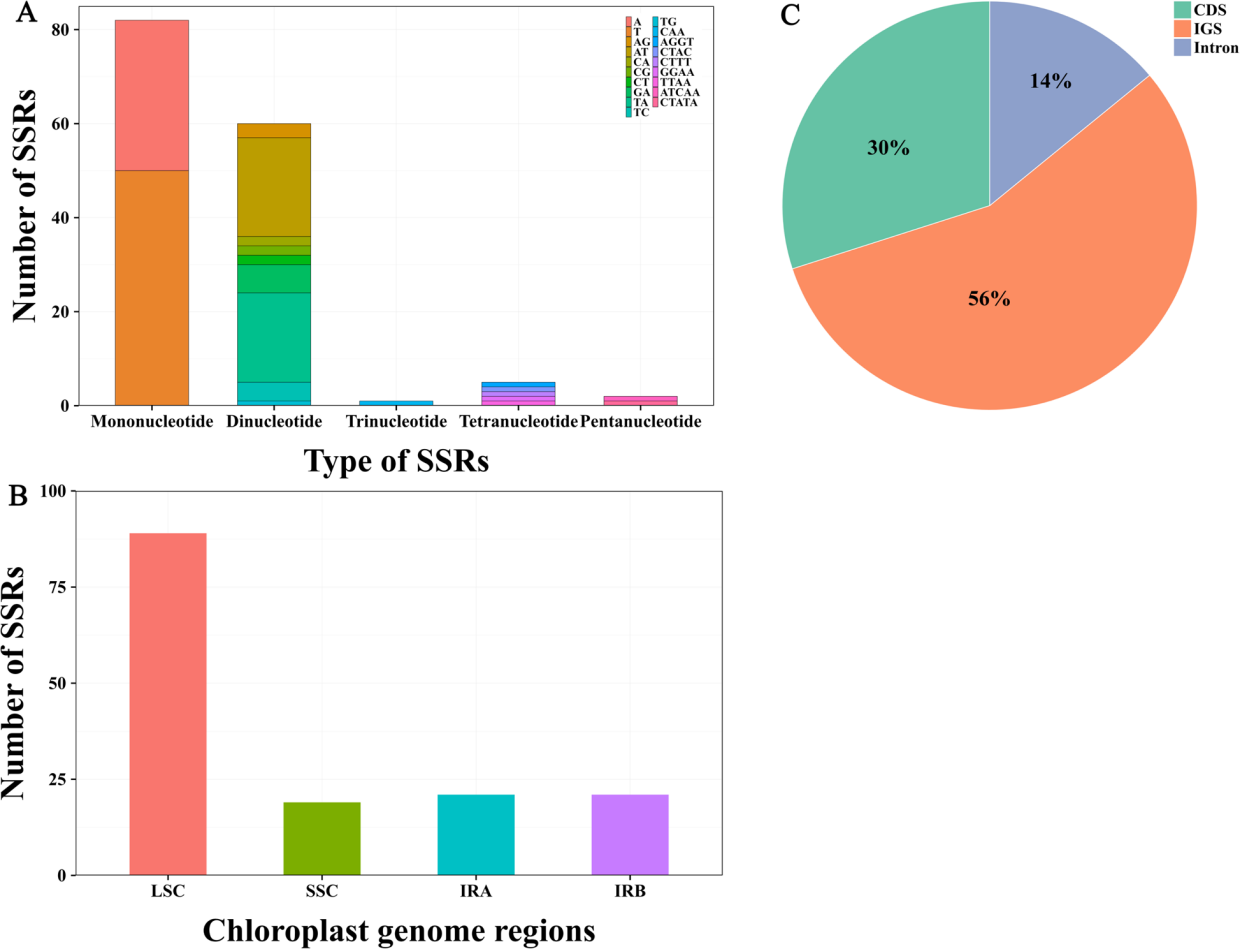
Examination of SSRs in the *Krascheninnikovia ceratoides* cp genome: **A** Categories and quantities of SSRs; **B** Placement of SSRs within the LSC, SSC, IRA, and IRB regions; **C** Localization of SSRs in coding sequence, IGS, and intron areas

The *K. ceratoides* cp genome features a variety of repeat sequences, comprising 47 forward repeats and 40 palindromic repeats, with no complementary or reverse repeats identified (Fig. 2A). The most frequent repeats were those ranging from 111 to 130 in length (33), followed by 79–90 (23), 91–110 (17), 151–162 (8), and the least common were 131–150 (6) (Fig. 2B). A total of 150 SSR loci were discovered in the *K. ceratoides* cp genome, spanning five SSR categories: mono-, di-, tri-, tetra-, and pentanucleotide repeats (Fig. 2A), with counts of 82, 60, 1, 5, and 2, respectively (Fig. 3A and Table S2). The distribution of these SSRs was as follows: 89 in the LSC region, 19 in the SSC region, 21 in the IRa region, and 21 in the IRb region (Fig. 3B). The majority (56%) of SSR loci were situated in intergenic spacer (IGS) regions, while only a small portion (14%) was found in intron regions (Fig. 3C).

**Fig. 4:**
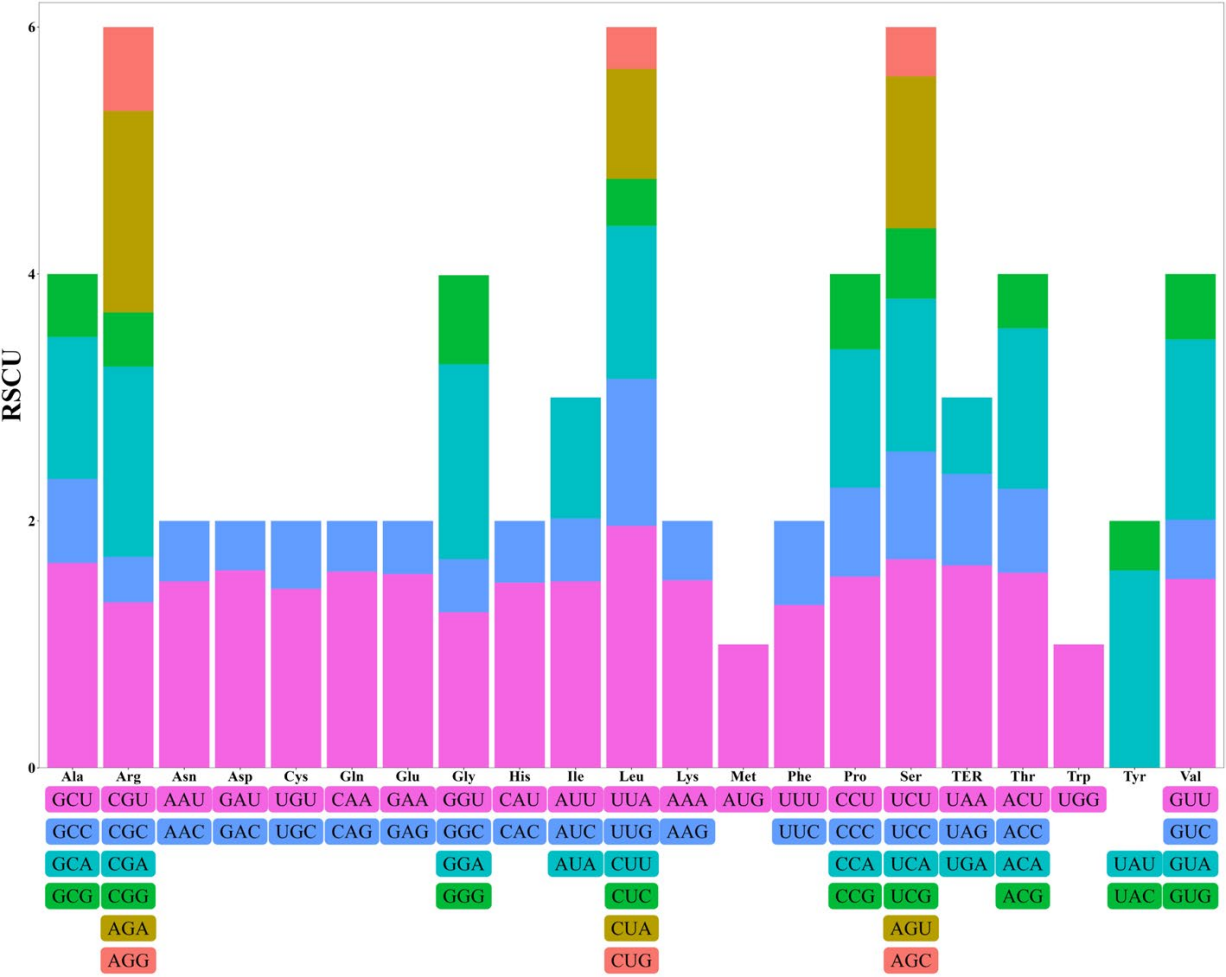
Codon usage biases in *Krascheninnikovia ceratoides* chloroplast genome

An examination of codon usage in the chloroplast genome of *K. ceratoides* identified 25,398 codons representing 20 amino acids and stop codons across the 68 PCGs. Leucine was found to be the most frequent codon, accounting for 10.53% of the total codons, followed by isoleucine (8.39%), serine (7.51%), and the least common was cysteine (1.17%) (Table S3). Additionally, a total of 30 degenerate codons were identified with Relative Synonymous Codon Usage (RSCU) values greater than one, indicating a bias in codon usage in *K. ceratoides*’ chloroplast genome. Of all the codons analyzed in *K. ceratoides* chloroplast genome, the AGA codon encoding arginine showed the strongest usage bias with a value of 1.96. Codons with RSCU values greater than 1.0 mostly ended with A or U at the third position, except for UUG (Fig. 4 and Table S3).

### Analysis of IR contraction and expansion

**Fig. 5:**
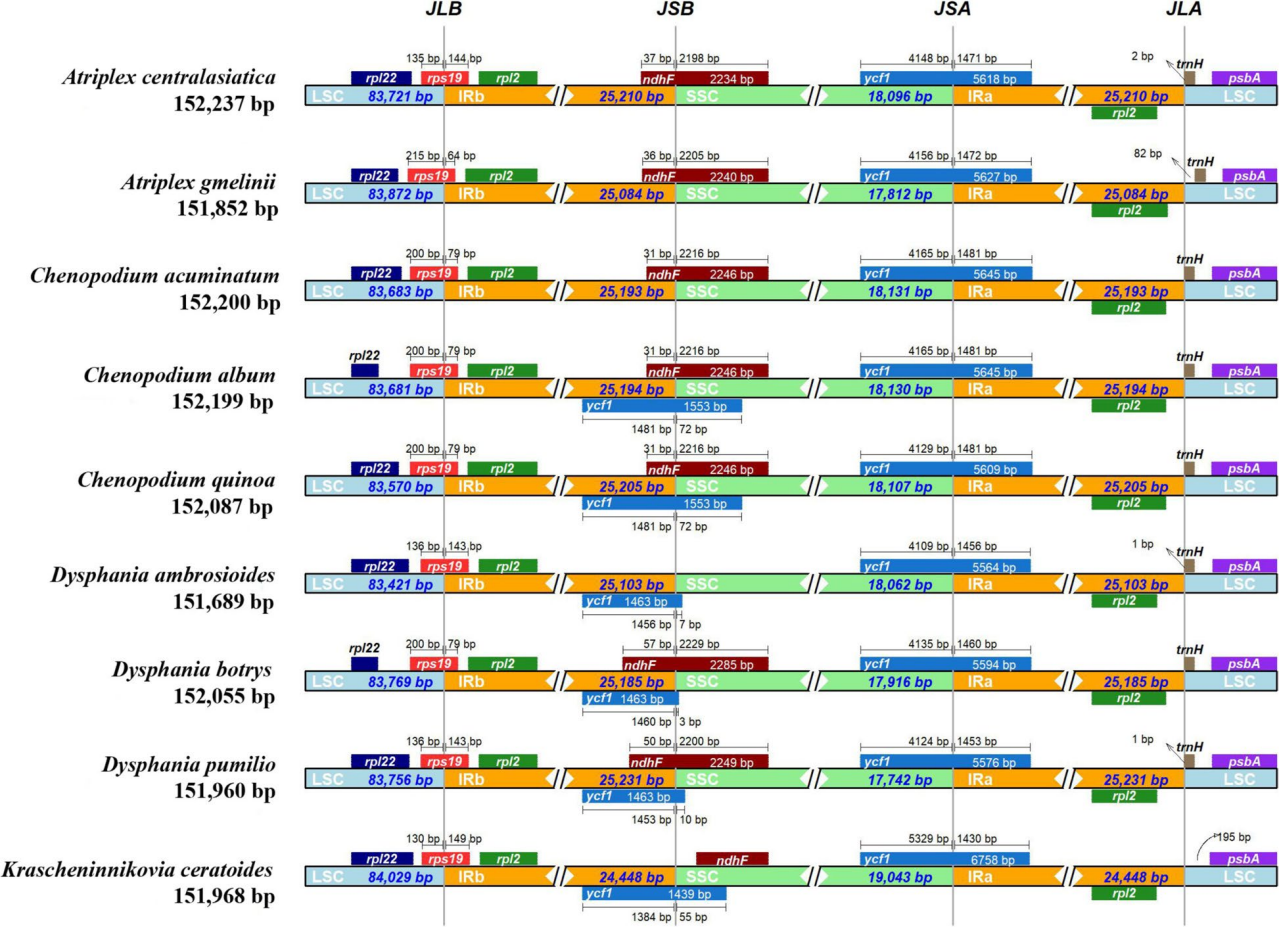
Analyze the boundaries between the LSC/SSC/IR regions and adjacent genes in Cyclolobeae (Amarathaceae), nine cp genomes were compared, including the cp genome of *Krascheninnikovia ceratoides*

Comparison of Cyclolobeae species cp genomes with that of *K. ceratoides* indicated that they had comparable numbers and positions of PCGs, tRNAs, and rRNAs, as well as comparable locations of the IR/LSC and IR/SSC boundary regions (Fig. 5). However, notable differences were detected, including expansions or contractions of the IR/LSC and IR/SSC boundary regions. In all Cyclolobeae cp genomes, the *rps19* gene crossed over the LSC/IRb region, but its length varied in the IRb region. The *ndhF* gene crossed over the IRb/SSC region in seven cp genomes, whereas in *K. ceratoides*, it was entirely located in the SSC region. The *ycf1* gene crossed over the SSC/IRa region junction in all Cyclolobeae cp genomes, with varying lengths located in the IRa region. Furthermore, the trnH gene was found in the LSC region of all cp genomes. These similarities and differences provide insights into the genetic diversity and evolution of Cyclolobeae species, including *K. ceratoides*.

### Comparative analysis of cp genomes in Cyclolobeae

**Fig. 6:**
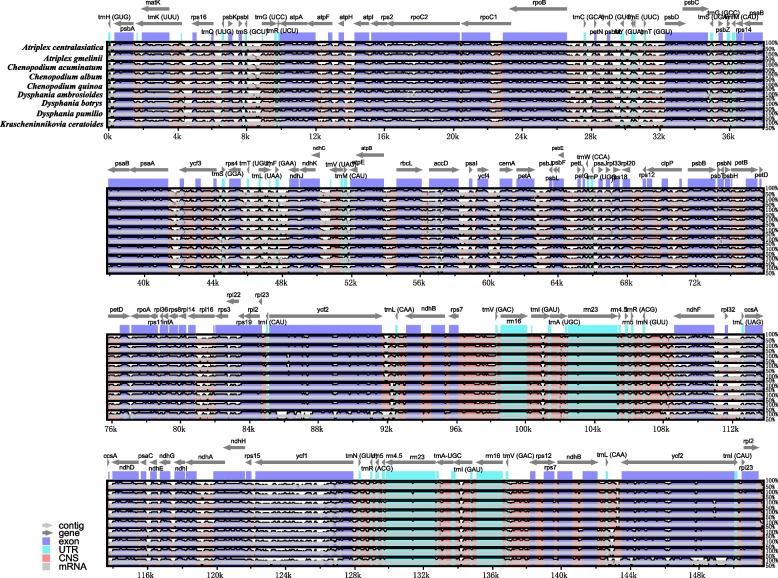
Multiple sequence alignment of nine Cyclolobeae chloroplast genomes

By aligning the cp genomes of nine Cyclolobeae specimens, it was observed that there are conserved structures shared among them. However, differences were detected, particularly in the non-coding regions and the single copy (SC) regions. The regions between genes that have a high degree of variation include *accD*-*psaI*, *ndhF*-*trnL*, *petA*-*psbJ*, *psbF*-*petL*, *trnC*-*psbM*, *trnS*-*trnG*, and *ycf2*-*trnL*. Moreover, protein-coding genes (PCGs) such as *accD*, *matK*, *ndhF*, *ndhK*, *ycf1*, and *ycf2* also demonstrated significant variation within their coding regions (Fig. 6).

**Fig. 7:**
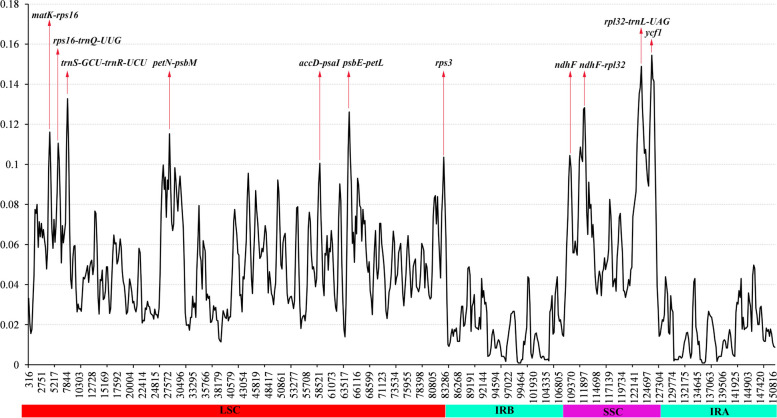
The analysis depicts the nucleotide diversity (Pi) of *Krascheninnikovia ceratoides* and three *Dysphania* species (*Dysphania ambrosioides*, *Dysphania botrys*, and *Dysphania pumilio*) based on their cp genomes

The analysis of nucleotide diversity among *K. ceratoides* and *Dysphania* species re-vealed an average Pi value of 0.04382 (Fig. 7). Nine highly variable regions with Pi values greater than 0.1 were identified, including *accD*-*psaI*, *rps3*, *psbE*-*petL*, *petN*-*psbM*, *rps16*-*trnQ*, *rpl32*-*trnL*, *ndhF*, *ndhF*-*rpl32*, *trnK*-*rps16*, *trnN*, *trnS*-*trnR*, and *ycf1* (Fig. 7). The LSC and SSC regions exhibited more nucleotide variability compared to the IR regions in both *K. ceratoides* and *Dysphania* species.

**Fig. 8:**
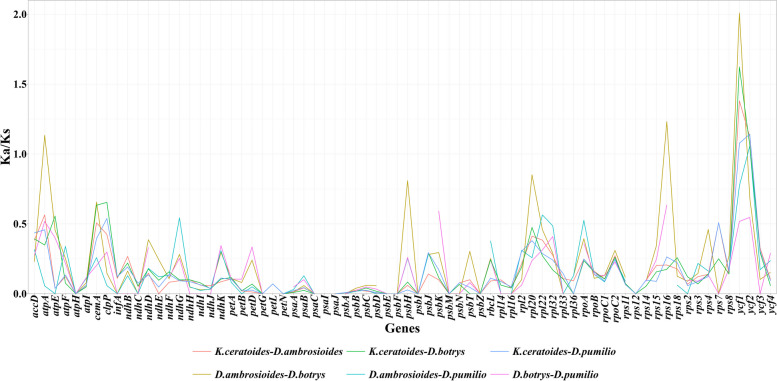
The protein coding genes of the cp genomes of *Krascheninnikovia ceratoides*, *Dysphania ambrosioides*, *Dysphania botrys*, and *Dysphania pumilio* were analyzed for their *K*a/*K*s substitution rates

The *K*a/*K*s substitution rates for 76 protein-coding genes in the cp genomes of *K. ceratoides* and *Dysphania* species were calculated, and the results showed that 17 genes had a *K*a/*K*s value of zero or could not be calculated. Out of the remaining 59 genes, four genes (*atpA*, *rps16*, *ycf1*, and *ycf2*) had *K*a/*K*s values higher than 1.0, while the *K*a/*K*s ratios of four other genes (*atpE*, *cemA*, *clpP*, *rps7*) were between 0.5 to 1.0 in both *K. ceratoides* and *Dysphania* species. Among these genes, *ycf1* and *ycf2* were detected in both *K. ceratoides* and *Dysphania* species, while *atpA* and *rps16* genes were only found in *D. ambrosioides* and *D. botrys* (Fig. 8).

### Phylogenetic analysis

**Fig. 9:**
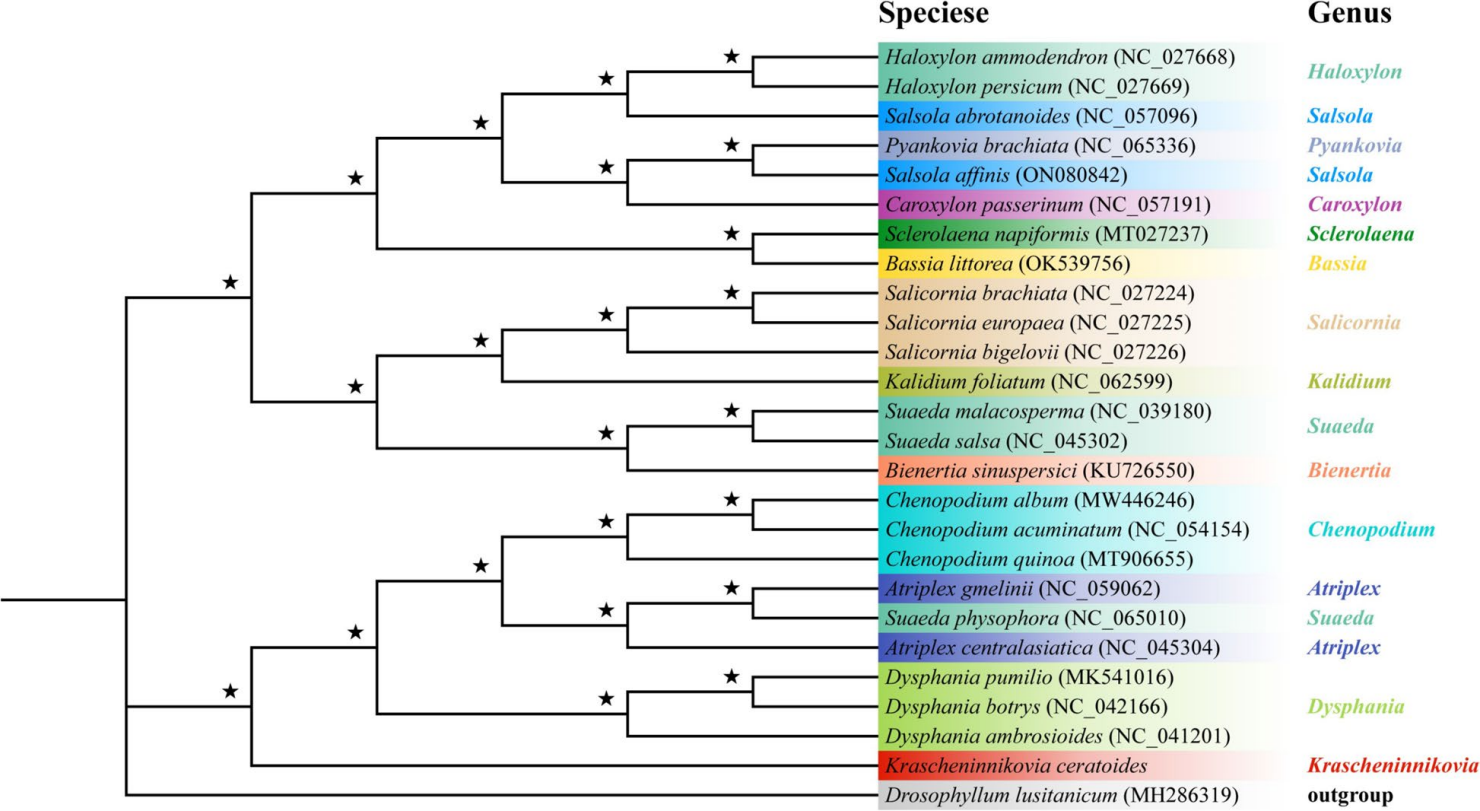
Phylogenetic tree was created based on the chloroplast genomes of 25 species from Amarathaceae, utilizing both BI and ML methods. The nodes with 100% bootstrap support and 1.00 pp. were marked with stars (“★”) indicating they were fully supported in both analyses

To construct phylogenetic trees for 25 Amarathaceae species, the ML and BI methods were employed, resulting in well-supported values for all major nodes and uniform topologies (see Fig. 9). The high support provided evidence that all 25 species formed a monophyletic group. Clade 1 was also monophyletic and comprised of *Bassia*, *Bienerita*, *Caroxylon*, *Haloxylon*, *Kalidium*, *Pyankovia*, *Salicornia*, *Salsola*, *Sclerolaena*, and Suaeda, which further split into two subclades: one containing *Bassia*, *Caroxylon*, *Haloxylon*, *Pyankovia*, *Salsola*, and *Sclerolaena*, and the other comprising. Clade 2 was also monophyletic and included *Atriplex*, *Bienerita*, *Kalidium*, *Salicornia*, and *Suaeda*, *Chenopodium*, *Dysphania*, *Suaeda*, and *Krascheninnikovia*. The analysis further revealed that *K. ceratoides* had a sister relationship with other species in Clade 2 (Fig. 9).

## Discussion

This study delves into the chloroplast (cp) genome of *K. ceratoides* from the Amarathaceae family. A comprehensive annotation and assembly process revealed insights into its taxonomic relationships and phylogenetic position. The identification of SSRs and genomic hotspots are pivotal for developing molecular markers, enhancing research in evolutionary history, population genetics, and breeding strategies for *K. ceratoides* and related species. The findings on nucleotide biases and amino acid representation in *K. ceratoides*’ cp genome are consistent with those in other eudicots, augmenting our understanding of genetic diversity and evolutionary connections within the Amarathaceae. This research sets a solid base for future studies on chloroplast genome evolution in this family, offering valuable tools for various genetic and ecological explorations.

The cp genome of *K. ceratoides* shows similarities with other angiosperm species. Like many other eudicot species, *K. ceratoides* has a trans-splicing *rps12* gene. The absence of the *ycf15* gene in the *K. ceratoides* cp genome is consistent with previous findings in species from unrelated orders, such as *Acorus*, *Ceratophyllum*, *Illicium*, and *Ranunculus*. The overall GC content of the *K. ceratoides* cp genome is 36.60%, which falls within the range reported for most species within its family. The LSC and SSC regions have lower GC content than the IR region. This difference can be attributed to the presence of eight GC-rich rRNA genes in the IR regions, as observed in previous studies. This genomic organization is typical of many angiosperm cp genomes and supports the idea that *K. ceratoides* is closely related to other members of its family.

Simple sequence repeats, or microsatellite sequences, are widely used as molecular markers in population genetics and evolutionary research due to their high degree of polymorphism at the species level. In the chloroplast genome of *K. ceratoides*, researchers have identified 150 SSR sites, which is a greater number than what has been found in other species within the Amarathaceae family. The study also revealed a greater abundance of mononucleotide SSRs compared to di-nucleotide and tri-nucleotide SSRs, which is consistent with previous findings in other Amarathaceae species [32]. SSRs with smaller units tend to exhibit increased polymorphism, making them more useful as molecular markers. As a result, the *K. ceratoides* SSRs, which predominantly consist of shorter units, have significant potential for the development of effective molecular markers. These markers could be employed in future population genetics and evolutionary studies, providing valuable insights into the biology and history of *K. ceratoides* and related species.

The finding that the amino acid cysteine is the least represented in the *K. ceratoides* cp genome is significant as it provides insights into the molecular characteristics and evolutionary relationships of this species with other eudicot plants. Cysteine’s low representation in the *K. ceratoides* cp genome is consistent with previous observations in other eudicot species [25, 26]. This similarity suggests that *K. ceratoides* shares common genomic features with other eudicots, which can contribute to our understanding of the evolutionary relationships among these plant species. Cysteine is an essential amino acid containing a sulfhydryl group, which can form disulfide bonds that play a crucial role in protein folding and stabilization. The low abundance of cysteine in the cp genome of *K. ceratoides* might have implications for the structure, function, and stability of cp proteins in this species. It could also potentially affect various biological processes, such as photosynthesis, that involve cp-encoded proteins. Further research on the functional roles of cysteine in *K. ceratoides* and its relationship with other amino acids in the cp genome could provide valuable insights into the molecular mechanisms and evolutionary forces shaping the biology of this species and related plants.

Relative Synonymous Codon Usage (RSCU) values were calculated to understand the usage bias of different codons for the same amino acid. 30 degenerate codons (codons that code for the same amino acid) showed RSCU values greater than one, indicating a preference for certain codons over others in the *K. ceratoides* cp genome. The AGA codon, which encodes arginine, displayed the highest usage bias with a value of 1.96. It is noteworthy that all other codons with RSCU values exceeding 1.0 ended in either A (adenine) or U (uracil), except for UUG. This observation suggests that the *K. ceratoides* cp genome has a bias towards using codons ending in A or U, which is a common pattern observed in chloroplast genomes. Understanding the codon usage patterns and biases in the *K. ceratoides* cp genome is important for studying gene expression, regulation, and evolution. This information can also be useful for comparative genomics and biotechnology applications, such as designing genes with optimized codon usage for efficient expression in this plant species.

The analysis revealed a preference for codons ending with A/U in the *K. ceratoides* cp genome. This finding is important because it provides insights into the molecular characteristics and evolutionary relationships of this species with other eudicot plants. The preference for A/U-ending codons in the *K. ceratoides* cp genome mirrors the A/U nucleotide bias observed in the cp genomes of other eudicots, such as Sinapis (*Brassicaceae* and *Leptodermis* (Rubiaceae) [33]. This similarity suggests that *K. ceratoides* shares common genomic features with other eudicot species, which can contribute to our understanding of the evolutionary relationships among these plant species. Codon usage bias, or the unequal usage of synonymous codons for the same amino acid, is a common feature in the genomes of various organisms. This bias can be influenced by factors such as mutational pressures, gene expression level, and selection for translational efficiency. The preference for A/U-ending codons in the *K. ceratoides* cp genome could have implications for the efficiency and accuracy of protein synthesis in this species, as well as the stability of its RNA structures. Further research on codon usage bias in *K. ceratoides* and its relationship with other molecular and evolutionary factors could provide valuable insights into the molecular mechanisms and evolutionary forces shaping the biology of this species and related plants. These findings further support the notion that *K. ceratoides* shares common genomic features with other eudicot species, providing insights into the evolutionary relationships and molecular characteristics of the cp genomes in this group of plants.

Variations in the IR regions, including contractions and expansions, are a common phenomenon in plant cp genomes and can result in differences in genome lengths. These variations can occur in both distantly and closely related species, leading to the generation of pseudogenes, gene duplications, and single-copy gene deletions. Compared to species from other families, such as *Lagerstroemia* of Lythraceae [34] and *Aconitum* of Ranunculaceae [35], the IR/SSC boundaries in Cyclolobeae are more conserved. Furthermore, the gene types and arrangements at the IR/SSC boundaries in the cp genome of Cyclolobeae are similar, with one notable exception: the loss of the *trnH* gene at the IRa/LSC boundary in *K. ceratoides*. This observation highlights the unique features of *K. ceratoides* cp genome, providing valuable insights into the evolution and genetic diversity within the Cyclolobeae group and the broader Amarathaceae family.

This study identified mutational hotspots for phylogenetic analyses and intraspecies discrimination at the species level through plastome-wide comparisons [36]. Even though the *rbcL* gene has been widely employed in phylogenetic research of the Amarathaceae family at the inter-familial level, its usefulness may be limited due to its relatively slower evolutionary rates. Instead, non-coding regions of the chloroplast genome may be more suitable for phylogenetic analysis as they tend to have higher mutation rates and greater variability, resulting from reduced selective pressures [37]. As a result, several non-coding regions, such as the intergenic spacers *atpB*-*rbcL*, *ndhF*-*rpL32*, *psbB*-*psbH*, and *trnL*-*trnF*, have been employed for phylogenetic reconstructions within Amarathaceae [38, 39].

The comparisons between the cp genome of *K. ceratoides* and other Amarathaceae species revealed sequence variations mainly within the SSC and LSC regions, with the LSC region being particularly divergent. Among the genes and non-coding regions examined, *accD*, *matK*, *ndhF*, *ndhK*, *ycf1*, and *ycf2* were identified as the most dissimilar, whereas the non-coding regions situated between *accD*-*psaI*, *ndhF*-*trnL*, *petA-psbJ*, *psbF-petL*, *trnC-psbM*, *trnS-trnG*, and *ycf2*-*trnL* exhibited the greatest degree of divergence. These genes and spacers can be considered as suitable candidates for developing molecular markers for resolving phylogenetic relationships and species delimitation within the Amarathaceae family.

It was observed that the SSC and LSC regions displayed more nucleotide variability compared to the IR regions in both *K. ceratoides* and *Dysphania* species. This finding aligns with the expectation that the IR regions tend to be more conserved as they play a crucial role in preserving the stability of the genome [40]. Understanding nucleotide diversity among plant species is important for studying their evolution, genetic structure, and adaptation. The identification of regions that exhibit high levels of variability can prove to be extremely valuable in the creation of molecular markers that can be utilized in population genetics, phylogenetics, and plant breeding. Additionally, this information can aid in the design of conservation strategies and the assessment of genetic resources in these species.

The *K*a/*K*s ratio in the cp genomes of *K. ceratoides* and *Dysphania* species, only two genes, *ycf1* and *ycf2*, showed values exceeding 1.0, indicating the presence of positive selective pressure. While *ycf1* and *ycf2* were initially identified as plastid-encoded proteins essential for the survival of *Chlamydomonas reinhardtii* and *Nicotiana tabacum*, their specific functions remain unclear [41]. However, numerous phylogenetic analyses of *ycf* genes in other species have suggested that positive selection acts on *ycf1* and *ycf2* [25, 26]. As a result, further investigation is necessary to determine the functional implications of positive selective pressure on these genes in the context of *K. ceratoides*, *Dysphania* species, and other related plants. Understanding the evolutionary forces shaping these genes may provide valuable insights into their functional roles in the biology and adaptation of these species.

The analysis of entire cp genomes in this study confirms that all 25 species of Amarathaceae form a monophyletic group. *K. ceratoides* is demonstrated to be closely related to *Atriplex*, *Chenopodium*, *Dysphania*, and *Suaeda*, which is consistent with previous studies based on nuclear ribosomal internal transcribed spacer and cp DNA data. However, it is important to acknowledge that future studies should include additional species to further support these relationships. Addressing taxonomic gaps in Amarathaceae is crucial for enhancing our understanding of the evolution and relationships within this diverse family. By incorporating more species and analyzing their cp genomes, researchers can gain a more comprehensive view of the phylogenetic relationships, molecular characteristics, and evolutionary history of the Amarathaceae family, contributing to a better understanding of this diverse group of plants.

## Conclusions

This study presents the first comprehensive chloroplast (cp) genome sequence of *K. ceratoides*, a halophytic semi-shrub of ecological, nutritional, and economic significance, that is widely distributed across arid regions of the Eurasian continent. By providing a thorough characterization of the cp genome, including its structure, gene content, and simple sequence repeats, this study expands the genomic resources available for the species. Moreover, it reports novel insights into the evolutionary relationships of *K. ceratoides* with other members of the Cyclolobeae (Amarathaceae) taxa, positioning it as a sister taxon to *Atriplex*, *Chenopodium*, *Dysphania*, and *Suaeda*. Additionally, the study uncovers evidence of positive selection pressure on *ycf1* and *ycf2* genes in *K. ceratoides*, which is an intriguing observation with potential implications for understanding the adaptive mechanisms of the species in desert ecosystems. The phylogenetic insights obtained from this research can be useful in addressing future taxonomic or nomenclatural challenges related to *K. ceratoides*. Moreover, the findings of this study provide a crucial resource for subsequent investigations into the genetic diversity and adaptation of *K. ceratoides* in arid and semi-arid regions across the Eurasian continent. This research contributes to a deeper understanding of the evolution and diversity within the *Krascheninnikovia* genus, paving the way for future studies on the ecology and utilization of *K. ceratoides*. The insights gained from this study offer fresh perspectives on the genetic features of *K. ceratoides*, establishing a foundation for future research focused on the plant’s classification, environmental impact, and potential applications.

## Materials & methods

### DNA extraction and sequencing

For this study, fresh leaves of *Krascheninnikovia ceratoides* were meticulously collected from Golmud City in Qinghai Province, China, located at coordinates 36.89° latitude and 93.14° longitude, with an altitude of 2845 m above sea level. The identification of the samples was conducted based on the morphological criteria outlined in “Flora of China” by Jinyuan Chen [10], ensuring accurate classification. To corroborate the authenticity of our specimens, voucher samples were diligently preserved and cataloged under the collection number QTP-LJQ-CHNR-020-1004. These voucher specimens are currently housed at the Herbarium of the Northwest Plateau Institute of Biology (HNWP), affiliated with the Chinese Academy of Sciences, situated in Xining, Qinghai Province, People’s Republic of China.

Total genomic DNA was extracted from the dried leaf tissue using a modified Cetyl Trimethyl Ammonium Bromide method [42] after drying the fresh leaves with silica gel. The quality of DNA samples was assessed through agarose gel electrophoresis, and qualified samples were fragmented using a Covaris Ultrasonic Crusher. The fragments were purified, repaired, and had poly-A tails and sequencing adaptors added before amplification using the NEB Next® Ultra™ DNA Library Prep Kit for Illumina (USA). The quality of DNA was assessed, and the samples were sequenced using the Illumina NovaSeq 6000 platform (Novegene Biological Information Technology Co., Ltd., Beijing, China).

### Genome assembly and annotation

The raw data was processed using FastQC v0.11.7 [43] and Fastp [44] to acquire high-quality clean reads by eliminating low-quality reads, shorter reads, and adapters. The high-quality reads were subsequently assembled into the complete cp genome of *K. ceratoides* using NOVOPlasty (version v4.2.1) [45] with the *Dysphania botrys* (NC_042166) cp genome as a reference. The complete cp genome was annotated using GeSeq [46] and PGA [47] by referencing the cp genomes of *Dysphania botrys* (NC_042166) and *Dysphania pumilio* (MK541016). The outcomes were manually examined and adjusted to minimize annotation errors, and the cp genome was submitted to GenBank with accession number OR635666. The cp genome’s gene map was visualized using the online tool Chloroplot (available at https://irscope.shinyapps.io/Chloroplot/) [48].

### Repeat sequence detection and codon usage analysis

The RE-Puter software [49] was employed to identify repeat sequences in the *K. ceratoides* cp genome, including forward repeat (F), reverse repeat (R), complementary repeat (C), and palindromic repeat (P). Detection parameters were set to a Hamming distance of 3, a minimum sequence identity of 90%, and a repeat size exceeding 30 bp. SSRs in the *K. ceratoides* cp genome were detected using the online tool MISA [50], with parameter settings of 1 = 9, 2 = 4, 3 = 4, 4 = 3, 5 = 3, 6 = 3 for mono-, di-, tri-, tetra-, penta-, and hexanucleotide sequences, respectively. PCGs with a minimum length of 200 bp were identified for codon usage analysis using CodonW version v1.4.2 [51] with default settings.

### Analysis of IR expansion, contraction, and comparative cp genome

To assess nucleotide diversity and selective pressure, the chloroplast genome of *K. ceratoides* was compared to eight other Cyclolobeae species (Amarathaceae) (https://www.ncbi.nlm.nih.gov) (Table S1). The similarities and differences of the LSC/IRb/SSC/IRa junctions among the nine cp genomes were visualized using IRscope [52] based on their annotations. Additionally, the *K. ceratoides* cp genome was compared to the eight Cyclolobeae species using the Shuffle-LAGAN alignment method within mVISTA [53], (available at http://genome.lbl.gov/vista/mvista/submit.html), with *Salicornia europaea* (NC_027225) used as a reference in mVISTA (Table S1) to identify highly variable regions.

### Nucleotide diversity and selective pressure analysis were performed on *K. ceratoides*’ chloroplast genome

The *K. ceratoides* cp genome was analyzed in comparison to three closely related *Dysphania* species to evaluate their nucleotide diversity and *K*a/*K*s ratio. The investigation centered on PCGs to identify synonymous (*K*s) and non-synonymous (*K*a) substitution rates. Nucleotide diversity (Pi) was computed using DnaSP V6.0 [54] with a 200 bp step size and a 600 bp window length [55]. The *K*a/*K*s ratio was calculated using *K*a*K*s_Calculator version 2.0 [56] employing the YN algorithm.

### Phylogenetic analysis

The goal was to build a phylogenetic tree for the Amarathaceae family. 24 cp genome sequences from 14 genera, representing two subfamilies within the family, were obtained and downloaded, along with the *Drosophyllum lusitanicum* (Drosophyllaceae) genome as an outgroup (Table S1). A multiple sequence alignment was generated using MAFFT version 7 [57] with default parameters, and any uncertainly aligned regions were removed using Gblocks version 0.91b [58].

The ML and BI methods were used to construct phylogenetic trees for 25 Amarathaceae species, which is a family of flowering plants. These methods are used to analyze the evolutionary relationships among species based on their genetic information.

The optimal DNA substitution model for *K. ceratoides* was determined using Mod-elFinder [59], which identified the TVM + F + I + G4 model. The phylogenetic analysis was conducted using the maximum-likelihood (ML) approach in IQ-TREE version 1.5.4 [60] with 5000 ultra-fast bootstrap iterations. Bayesian analysis (BI) was also performed using MrBayes version 3.2.6 [61], with two separate Markov Chain Monte Carlo chains executed for 2 million cycles, and sampling at every 1000 cycles. The convergence of the chains was indicated by an average split frequency of 0.01, and the initial 25% of all cycles were discarded as burn-in. The resulting trees were combined into a maximum clade credibility (MCC) tree.

### Supplementary Information

**Additional file 1: Table S1.** GenBank accession numbers for the cp genome of 25 species from Amarathaceae and one outgroup used in this study. **Table S2.** Summary of SSRs in the cp genome of *Krascheninnikovia ceratoides*. **Table S3.** Relative synonymous codon usage of each amino acid in *Krascheninnikovia ceratoides.*

## Data Availability

All data generated or analyzed during the present study are included in this published article. The raw sequencing data have been deposited in the Sequence Read Archive (SRA) of NCBI with accession number SRR26283322.
