# Supplementary material for: Comparative analysis and characterization of the chloroplast genome of Krascheninnikovia ceratoides (Amarathaceae): a xerophytic semi-shrub exhibiting drought resistance and high-quality traits
Source: BMC Genom Data. 2024 Jan 29;25:10. doi: 10.1186/s12863-024-01197-y (PMC10826016; doi:10.1186/s12863-024-01197-y)
Supplement: Supplementary file 1 — Additional file 1: Table S1. GenBank accession numbers for the cp genome of 25 species from Amarathaceae and one outgroup used in this study. Table S2. Summary of SSRs in the cp genome of Krascheninnikovia ceratoides. Table S3. Relative synonymous codon usage of each amino acid in Krascheninnikovia ceratoides. [file 12863_2024_1197_MOESM1_ESM.docx]

**Table S1** GenBank accession numbers for the chloroplast genome of 25 species from Chenopodiaceae and one outgroup used in this study.

| Family | Genus | Species | Accession number of GenBank |
| --- | --- | --- | --- |
| Chenopodiaceae | *Krascheninnikovia* | *Krascheninnikovia ceratoides* | OR635666 |
|  | *Dysphania* | *Dysphania ambrosioides* | NC_041201 |
|  |  | *Dysphania botrys* | NC_042166 |
|  |  | *Dysphania pumilio* | MK541016 |
|  | *Atriplex* | *Atriplex centralasiatica* | NC_045304 |
|  |  | *Atriplex gmelinii* | NC_059062 |
|  | *Suaeda* | *Suaeda phyophora* | NC_065010 |
|  | *Chenopodium* | *Chenopodium acuminatum* | NC_054154 |
|  |  | *Chenopodium album* | MW446246 |
|  |  | *Chenopodium quinosa* | MT906655 |
|  | *Bienerita* | *Bienerita sinuspersici* | KU726550 |
|  | *Suaeda* | *Suaeda salsa* | NC_045302 |
|  |  | *Suaeda malacosperma* | NC_039180 |
|  | *Kalidium* | *Kalidium foliatum* | NC_062599 |
|  | *Salicornia* | *Salicornia bigelovii* | NC_027226 |
|  |  | *Salicornia europaea* | NC_027225 |
|  |  | *Salicornia brachiata* | NC_027224 |
|  | *Bassia* | *Bassia littorea* | OK539756 |
|  | *Sclerolaena* | *Sclerolaena napiformis* | MT027237 |
|  | *Caroxylon* | *Caroxylon passerinum* | NC_057191 |
|  | *Salsola* | *Salsola affinis* | ON080842 |
|  |  | *Salsola abrotanoides* | NC_057096 |
|  | *Pyankovia* | *Pyankovia brachiata* | NC_065336 |
|  | *Haloxylon* | *Haloxylon persicum* | NC_027669 |
|  |  | *Haloxylon ammodendron* | NC_027668 |
| Drosophyllaceae | *Drosophyllum* | *Drosophyllum lusitanicum* | MH286319 |

**Table S2** Summary of simple sequence repeats in the chloroplast genome of *Krascheninnikovia ceratoides*.

| No. | SSRs | Start | End | Location |
| --- | --- | --- | --- | --- |
| 1 | (A)9 | 3456 | 3464 | IGS |
| 2 | (AT)5 | 4424 | 4433 | IGS |
| 3 | (TA)4 | 5003 | 5010 | Intron (*rps16*) |
| 4 | (T)11gacaataaaagcgacatccctaa  Aaaagaaaaatccatgtttctttttgagctcagacattca(T)9 | 6051 | 6133 | IGS |
| 5 | (A)14 | 6892 | 6905 | IGS |
| 6 | (A)10 | 7517 | 7526 | IGS |
| 7 | (TA)4atgat(A)9tgcttattattaaag  caaaaacacaataataaaatagaaccaac  atgacatttttttcgtttctttcccctttatt(CTATA)3 | 8245 | 8357 | IGS |
| 8 | (T)10(AG)4 | 8860 | 8877 | IGS |
| 9 | (T)9 | 11129 | 11137 | IGS |
| 10 | (A)10(TA)5aatatagttgctaagtataa  gtaattgtcagcaaagttg(T)10cttcaaa  atccaaaaattttgcttactttaatacgt  aggtcatcgactcagcctttggcacttatttactgaa(T)9 | 11841 | 11991 | Intron (*atpF*) |
| 11 | (T)9 | 12665 | 12673 | IGS |
| 12 | (GGAA)3 | 12802 | 12813 | IGS |
| 13 | (CA)4 | 13271 | 13278 | IGS |
| 14 | (T)10 | 15745 | 15754 | IGS |
| 15 | (T)10 | 16066 | 16075 | CDS (*rpoC2*) |
| 16 | (T)9 | 17777 | 17785 | CDS (*rpoC2*) |
| 17 | (T)13 | 17941 | 17953 | CDS (*rpoC2*) |
| 18 | (AT)4 | 18528 | 18535 | CDS (*rpoC2*) |
| 19 | (TA)4 | 19294 | 19301 | CDS (*rpoC2*) |
| 20 | (T)10 | 22033 | 22042 | Intron (*rpoC1*) |
| 21 | (A)9gaagtc(T)9 | 22365 | 22388 | Intron (*rpoC2*) |
| 22 | (TA)4 | 26636 | 26643 | IGS |
| 23 | (CAA)4 | 27662 | 27673 | IGS |
| 24 | (CTTT)3 | 28448 | 28459 | IGS |
| 25 | (A)9 | 28748 | 28756 | IGS |
| 26 | (A)9 | 30786 | 30794 | IGS |
| 27 | (TA)4atataaagtgaaactaatattca  Ttctattataattgtaatgataagttgttta  tcgaatcagcaactattccctattattctatta(T)9atatta(T)11 | 31178 | 31298 | IGS |
| 28 | (T)9 | 31434 | 31442 | IGS |
| 29 | (A)9 | 31585 | 31593 | IGS |
| 30 | (GA)4 | 34559 | 34566 | CDS (*trnS-UGA*) |
| 31 | (A)11 | 34848 | 34858 | IGS |
| 32 | (A)9 | 35717 | 35725 | IGS |
| 33 | (A)10 | 36433 | 36442 | IGS |
| 34 | (TA)4 | 41487 | 41494 | IGS |
| 35 | (TA)4 | 42429 | 42436 | Intron (*ycf3*) |
| 36 | (A)12 | 43099 | 43110 | Intron (ycf3) |
| 37 | (TA)4 | 44713 | 44720 | IGS |
| 38 | (TA)4catcatatacaaaccctt(TA)4  atattattatatacaataatatataatattat(TA)5 | 45700 | 45775 | IGS |
| 39 | (AT)4 | 46448 | 46455 | IGS |
| 40 | (T)10gcttttg(A)10 | 46818 | 46844 | IGS |
| 41 | (T)10 | 47025 | 47034 | Intron (*trnL-UAA*) |
| 42 | (A)10 | 47215 | 47224 | Intron (*trnL-UAA*) |
| 43 | (T)9 | 48040 | 48048 | IGS |
| 44 | (T)10 | 49804 | 49813 | IGS |
| 45 | (TA)4 | 50441 | 50448 | IGS |
| 46 | (T)9 | 50914 | 50922 | IGS |
| 47 | (T)13 | 51136 | 51148 | Intron (*trnV-UAC*) |
| 48 | (TC)4gttctgctt(A)9 | 51350 | 51375 | Intron (*trnV-UAC*) |
| 49 | (ATCAA)3 | 51495 | 51509 | Intron (*trnV-UAC*) |
| 50 | (T)11 | 52077 | 52087 | IGS |
| 51 | (T)9 | 54049 | 54057 | CDS (*atpE*) |
| 52 | (T)9 | 54289 | 54297 | IGS |
| 53 | (AT)4 | 54681 | 54688 | IGS |
| 54 | (GA)4 | 55362 | 55369 | CDS (*rbcL*) |
| 55 | (TA)4ttagaggagattctac(A)9 | 56638 | 56670 | IGS |
| 56 | (A)9 | 60689 | 60697 | IGS |
| 57 | (AT)4 | 61725 | 61732 | CDS (*petA*) |
| 58 | (TA)4 | 62782 | 62789 | IGS |
| 59 | (A)11gaacaaaaaatggattaattg  attttattttacttaatccatatcca(T)9 | 64759 | 64825 | IGS |
| 60 | (A)11 | 65127 | 65137 | IGS |
| 61 | (T)10 | 65253 | 65262 | IGS |
| 62 | (T)13 | 65734 | 65746 | IGS |
| 63 | (A)9 | 66508 | 66516 | IGS |
| 64 | (A)9 | 67050 | 67058 | IGS |
| 65 | (T)10ctatatttatat  taaatattatat(TA)4 | 67538 | 67579 | IGS |
| 66 | (A)16 | 69952 | 69967 | IGS |
| 67 | (AT)5 | 76909 | 76918 | Intron (*petD*) |
| 68 | (T)11 | 77799 | 77809 | CDS (*rpoA*) |
| 69 | (GA)4 | 78486 | 78493 | IGS |
| 70 | (A)9 | 80861 | 80869 | IGS |
| 71 | (T)11 | 82352 | 82362 | Intron (*rpl16*) |
| 72 | (T)9 | 83807 | 83815 | CDS (*rpl22*) |
| 73 | (T)9 | 84156 | 84164 | CDS (*rps19*) |
| 74 | (AT)4tgaataaattaattatata(AT)4 | 85492 | 85526 | IGS |
| 75 | (AT)4tgaataaattaattatata(AT)4(TA)4 | 85654 | 85696 | IGS |
| 76 | (GA)4tatt(GA)4 | 86151 | 86170 | CDS (*ycf2*) |
| 77 | (AT)4 | 87626 | 87633 | CDS (*ycf3*) |
| 78 | (A)9catgcttttttg(GA)4 | 88246 | 88274 | CDS (*ycf4*) |
| 79 | (AT)4 | 92439 | 92446 | IGS |
| 80 | (AG)4 | 92966 | 92973 | CDS (*ndhB*) |
| 81 | (T)9 | 101800 | 101808 | Intron(*trnA-UGC*) |
| 82 | (CG)4 | 103380 | 103387 | CDS (*rrn23*) |
| 83 | (AGGT)3agagaatacctaggggcgcgagacaa(CT)4 | 103797 | 103842 | CDS (*rrn23*) |
| 84 | (T)9 | 106116 | 106124 | IGS |
| 85 | (TG)4 | 107086 | 107093 | IGS |
| 86 | (A)10cagcttaagcaatgaattttc  taagagaattacaagtctagacaaa  ggactttcttatatagatgtactcg(A)9 | 108286 | 108375 | CDS (*ycf1*) |
| 87 | (A)9 | 108542 | 108550 | CDS (*ndhF*) |
| 88 | (TA)4 | 108821 | 108828 | CDS (*ndhF*) |
| 89 | (AT)4 | 110588 | 110595 | CDS (*ndhF*) |
| 90 | (TTAA)3 | 110830 | 110841 | IGS |
| 91 | (T)13 | 112458 | 112470 | IGS |
| 92 | (T)9 | 112574 | 112582 | IGS |
| 93 | (T)9 | 116039 | 116047 | IGS |
| 94 | (T)11atgtgtaaagaaaagaatag  ttcggaactaaaactatttaaaatcatta  gagaaaacatattttaaatacaaa  caaagtttcgattttcaccaatc(A)9 | 117479 | 117594 | IGS |
| 95 | (T)9 | 119182 | 119190 | Intron (*ndhA*) |
| 96 | (AT)4 | 122220 | 122227 | IGS |
| 97 | (T)12ctttataagttttttatcggattc  cgtttccgtagaatcttcttc(T)11cc  tttataggttttgttatatcagatcctatggaatcttcttcaa(T)9 | 122921 | 123042 | CDS (*ycf1*) |
| 98 | (T)14 | 124145 | 124158 | CDS (*ycf1*) |
| 99 | (AT)4 | 124767 | 124774 | CDS (*ycf1*) |
| 100 | (T)9 | 125002 | 125010 | CDS (*ycf1*) |
| 101 | (T)11aaaaatgtaaggatttcaatttgaaactttcgaagaaa(T)9 | 125164 | 125221 | CDS (*ycf1*) |
| 102 | (T)9cgagtacatctatataagaaagtc  ctttgtctagacttgtaattctctta  gaaaattcattgcttaagctg(T)10 | 127669 | 127758 | CDS (*ycf1*) |
| 103 | (CA)4 | 128951 | 128958 | IGS |
| 104 | (A)9 | 129874 | 129882 | IGS |
| 105 | (AG)4ttgtctcgcgcccctaggtattct(CTAC)3 | 132156 | 132199 | CDS (*rrn23*) |
| 106 | (CG)4 | 132611 | 132618 | CDS (*rrn23*) |
| 107 | (A)9 | 134190 | 134198 | Intron (*trnA-UGC*) |
| 108 | (CT)4 | 143025 | 143032 | CDS (*ndhB*) |
| 109 | (TA)4 | 143551 | 143558 | IGS |
| 110 | (TC)4caaaaaagcatg(T)9 | 147724 | 147752 | CDS (*ycf2*) |
| 111 | (AT)4 | 148365 | 148372 | CDS (*ycf2*) |
| 112 | (TC)4aata(TC)4 | 149828 | 149847 | CDS (*ycf2*) |
| 113 | (AT)4a(AT)4tatataattaatttattca(AT)4 | 150301 | 150344 | IGS |
| 114 | (AT)4tatataattaatttattca(AT)4 | 150472 | 150506 | IGS |
| 115 | (A)9 | 151834 | 151842 | IGS |

**Table S3** Relative synonymous codon usage of each amino acid in *Krascheninnikovia ceratoides*.

| Amino acid | Codon | Number | RSCU | Ratio (%) | Amino acid | Codon | Number | RSCU | Ratio (%) |
| --- | --- | --- | --- | --- | --- | --- | --- | --- | --- |
| Phe | UUU | 1004 | 1.32 | 5.98 | Ala | GCU | 558 | 1.66 | 5.29 |
|  | UUC | 514 | 0.68 |  |  | GCC | 228 | 0.68 |  |
| Leu | UUA | 874 | 1.96 | 10.53 |  | GCA | 387 | 1.15 |  |
|  | UUG | 530 | 1.19 |  |  | GCG | 170 | 0.51 |  |
|  | CUU | 553 | 1.24 |  | TER^*^ | UAA | 40 | 1.64 | 0.29 |
|  | CUC | 170 | 0.38 |  |  | UAG | 18 | 0.74 |  |
|  | CUA | 396 | 0.89 |  |  | UGA | 15 | 0.62 |  |
|  | CUG | 152 | 0.34 |  | His | CAU | 444 | 1.50 | 2.33 |
| Ile | AUU | 1071 | 1.51 | 8.39 |  | CAC | 147 | 0.50 |  |
|  | AUC | 363 | 0.51 |  | Gln | CAA | 732 | 1.59 | 3.63 |
|  | AUA | 697 | 0.98 |  |  | CAG | 191 | 0.41 |  |
| Met | AUG | 557 | 1.00 | 2.19 | Asn | AAU | 914 | 1.51 | 4.76 |
| Val | GUU | 517 | 1.53 | 5.32 |  | AAC | 294 | 0.49 |  |
|  | GUC | 162 | 0.48 |  | Lys | AAA | 1105 | 1.52 | 5.71 |
|  | GUA | 492 | 1.46 |  |  | AAG | 346 | 0.48 |  |
|  | GUG | 179 | 0.53 |  | Asp | GAU | 839 | 1.60 | 4.13 |
| Ser | UCU | 537 | 1.69 | 7.51 |  | GAC | 209 | 0.40 |  |
|  | UCC | 278 | 0.87 |  | Glu | GAA | 1142 | 1.57 | 5.74 |
|  | UCA | 394 | 1.24 |  |  | GAG | 317 | 0.43 |  |
|  | UCG | 181 | 0.57 |  | Cys | UGU | 216 | 1.45 | 1.17 |
|  | AGU | 393 | 1.23 |  |  | UGC | 82 | 0.55 |  |
|  | AGC | 123 | 0.40 |  | Trp | UGG | 421 | 1.00 | 1.66 |
| Pro | CCU | 408 | 1.55 | 4.15 | Arg | CGU | 331 | 1.34 | 5.84 |
|  | CCC | 190 | 0.72 |  |  | CGC | 92 | 0.37 |  |
|  | CCA | 295 | 1.12 |  |  | CGA | 380 | 1.54 |  |
|  | CCG | 161 | 0.61 |  |  | CGG | 110 | 0.44 |  |
| Thr | ACU | 524 | 1.58 | 5.22 |  | AGA | 404 | 1.63 |  |
|  | ACC | 224 | 0.68 |  |  | AGG | 167 | 0.68 |  |
|  | ACA | 432 | 1.30 |  | Gly | GGU | 524 | 1.26 | 6.54 |
|  | ACG | 147 | 0.44 |  |  | GGC | 178 | 0.43 |  |
| Tyr | UAU | 735 | 1.60 | 3.61 |  | GGA | 658 | 1.58 |  |
|  | UAC | 183 | 0.40 |  |  | GGG | 301 | 0.72 |  |

Note: *represents termination codon.
